# 2-Acetyl-3-methyl­pyrazine phenyl­sulfonyl­hydrazone

**DOI:** 10.1107/S1600536808009331

**Published:** 2008-04-10

**Authors:** Xi-Shi Tai, Yi-Min Feng, Fan-Yuan Kong

**Affiliations:** a Department of Chemistry and Chemical Engineering, Weifang University, Weifang 261061, People’s Republic of China

## Abstract

In the title compound, C_13_H_14_N_4_O_2_S, the dihedral angle between the aromatic rings is 55.42 (14)°. In the crystal structure, an N—H⋯O hydrogen bond leads to chains of mol­ecules along [001].

## Related literature

For related literature, see: Tai *et al.* (2008 ▶).
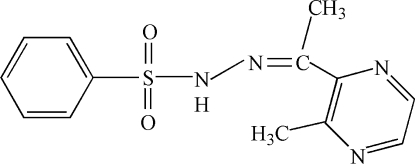


## Experimental

### 

#### Crystal data

C_13_H_14_N_4_O_2_S
*M* *_r_* = 290.34
Monoclinic, 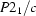

*a* = 10.9848 (15) Å
*b* = 16.7921 (18) Å
*c* = 7.4817 (10) Å
β = 97.264 (1)°
*V* = 1369.0 (3) Å^3^
*Z* = 4
Mo *K*α radiation
μ = 0.24 mm^−1^
*T* = 298 (2) K
0.50 × 0.28 × 0.14 mm

#### Data collection

Bruker SMART CCD diffractometer
Absorption correction: multi-scan (*SADABS*; Bruker, 2000 ▶) *T*_min_ = 0.888, *T*_max_ = 0.967
6807 measured reflections
2402 independent reflections
1499 reflections with *I* > 2σ(*I*)
*R*_int_ = 0.061

#### Refinement

*R*[*F* ^2^ > 2σ(*F* ^2^)] = 0.046
*wR*(*F* ^2^) = 0.103
*S* = 1.02
2402 reflections
183 parameters
H-atom parameters constrained
Δρ_max_ = 0.32 e Å^−3^
Δρ_min_ = −0.31 e Å^−3^

### 

Data collection: *SMART* (Bruker, 2000 ▶); cell refinement: *SAINT* (Bruker, 2000 ▶); data reduction: *SAINT*; program(s) used to solve structure: *SHELXS97* (Sheldrick, 2008 ▶); program(s) used to refine structure: *SHELXL97* (Sheldrick, 2008 ▶); molecular graphics: *SHELXTL* (Sheldrick, 2008 ▶); software used to prepare material for publication: *SHELXTL*.

## Supplementary Material

Crystal structure: contains datablocks global, I. DOI: 10.1107/S1600536808009331/hb2717sup1.cif

Structure factors: contains datablocks I. DOI: 10.1107/S1600536808009331/hb2717Isup2.hkl

Additional supplementary materials:  crystallographic information; 3D view; checkCIF report

## Figures and Tables

**Table 1:** Hydrogen-bond geometry (Å, °)

*D*—H⋯*A*	*D*—H	H⋯*A*	*D*⋯*A*	*D*—H⋯*A*
N1—H1⋯O2^i^	0.86	2.34	3.027 (3)	137

Symmetry code: (i) 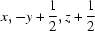.

**Fig. 1.**
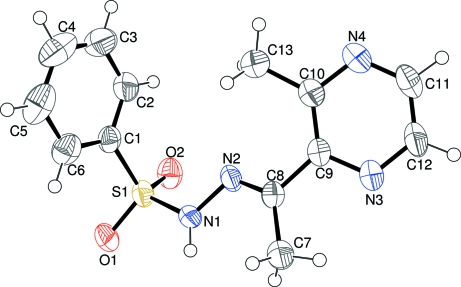
The molecular structure of (I) showing 30% displacement ellipsoids for the non-hydrogen atoms.

## supplementary crystallographic information

### Comment

As part of our ongoing studies of aroylhydrazones as possible ligands (Tai *et al.*, 2008), we now report the synthesis and structure of the title compound, (I), (Fig. 1).

The dihedral angle between the aromatic ring planes is 55.42 (14)°. Otherwise, the geometrical parameters for (I) are normal. In the crystal of (I), an N-H···O hydrogen bond (Table 1) leads to [001] chains.

### Experimental

1 mmol of 2-Acetyl-3-methylpyrazine (1 mmol) was added to a solution of benzenesulfonyl hydrazide (1 mmol) in 5 ml of 95% ethanol. The mixture was continuously stirred for 4 h at refluxing temperature, evaporating some ethanol, then, upon cooling, the solid product was collected by filtration and dried *in vacuo* (yield 67%). Clear blocks of (I) were obtained by evaporation from a methanol solution after 3 days.

### Refinement

The H atoms were placed geometrically (C—H = 0.93–0.96 Å, N—H = 0.86 Å) and refined as riding with *U*_iso_(H) = 1.2*U*_eq_(carrier) or 1.5*U*_eq_(methyl C).

### Crystal data

**Table:**

C_13_H_14_N_4_O_2_S	*F*_000_ = 608
*M**_r_* = 290.34	*D*_x_ = 1.409 Mg m^−^^3^
Monoclinic, *P*2_1_/*c*	Mo *K*α radiation λ = 0.71073 Å
Hall symbol: -P 2ybc	Cell parameters from 1439 reflections
*a* = 10.9848 (15) Å	θ = 3.0–22.9º
*b* = 16.7921 (18) Å	µ = 0.24 mm^−^^1^
*c* = 7.4817 (10) Å	*T* = 298 (2) K
β = 97.264 (1)º	Block, colourless
*V* = 1369.0 (3) Å^3^	0.50 × 0.28 × 0.14 mm
*Z* = 4	

### Data collection

**Table:**

Bruker SMART CCD diffractometer	2402 independent reflections
Radiation source: fine-focus sealed tube	1499 reflections with *I* > 2σ(*I*)
Monochromator: graphite	*R*_int_ = 0.061
*T* = 298(2) K	θ_max_ = 25.0º
ω scans	θ_min_ = 1.9º
Absorption correction: multi-scan(SADABS; Bruker, 2000)	*h* = −13→8
*T*_min_ = 0.888, *T*_max_ = 0.967	*k* = −19→17
6807 measured reflections	*l* = −8→8

### Refinement

**Table:**

Refinement on *F*^2^	Secondary atom site location: difference Fourier map
Least-squares matrix: full	Hydrogen site location: inferred from neighbouring sites
*R*[*F*^2^ > 2σ(*F*^2^)] = 0.046	H-atom parameters constrained
*wR*(*F*^2^) = 0.103	*w* = 1/[σ^2^(*F*_o_^2^) + (0.036*P*)^2^] where *P* = (*F*_o_^2^ + 2*F*_c_^2^)/3
*S* = 1.03	(Δ/σ)_max_ < 0.001
2402 reflections	Δρ_max_ = 0.32 e Å^−^^3^
183 parameters	Δρ_min_ = −0.31 e Å^−^^3^
Primary atom site location: structure-invariant direct methods	Extinction correction: none

### Special details

**Table:**

Geometry. All e.s.d.'s (except the e.s.d. in the dihedral angle between two l.s. planes) are estimated using the full covariance matrix. The cell e.s.d.'s are taken into account individually in the estimation of e.s.d.'s in distances, angles and torsion angles; correlations between e.s.d.'s in cell parameters are only used when they are defined by crystal symmetry. An approximate (isotropic) treatment of cell e.s.d.'s is used for estimating e.s.d.'s involving l.s. planes.
Refinement. Refinement of *F*^2^ against ALL reflections. The weighted *R*-factor *wR* and goodness of fit *S* are based on *F*^2^, conventional *R*-factors *R* are based on *F*, with *F* set to zero for negative *F*^2^. The threshold expression of *F*^2^ > σ(*F*^2^) is used only for calculating *R*-factors(gt) *etc*. and is not relevant to the choice of reflections for refinement. *R*-factors based on *F*^2^ are statistically about twice as large as those based on *F*, and *R*- factors based on ALL data will be even larger.

### Fractional atomic coordinates and isotropic or equivalent isotropic displacement parameters (Å^2^)

**Table:**

	*x*	*y*	*z*	*U*_iso_*/*U*_eq_	
N1	0.76385 (18)	0.24028 (12)	0.7039 (3)	0.0401 (6)	
H1	0.7785	0.2885	0.7389	0.048*	
N2	0.64583 (19)	0.21106 (12)	0.6591 (3)	0.0386 (6)	
N3	0.3511 (2)	0.28276 (13)	0.5461 (3)	0.0504 (7)	
N4	0.2852 (2)	0.12465 (14)	0.5560 (3)	0.0552 (7)	
O1	0.98156 (16)	0.21805 (11)	0.7451 (3)	0.0528 (6)	
O2	0.85153 (17)	0.14561 (10)	0.5061 (2)	0.0508 (5)	
S1	0.87236 (6)	0.17593 (4)	0.68486 (10)	0.0412 (2)	
C1	0.8548 (2)	0.09773 (15)	0.8320 (4)	0.0385 (7)	
C2	0.7922 (3)	0.03023 (16)	0.7692 (4)	0.0539 (8)	
H2	0.7631	0.0249	0.6475	0.065*	
C3	0.7734 (3)	−0.02929 (19)	0.8895 (6)	0.0704 (10)	
H3A	0.7318	−0.0754	0.8494	0.084*	
C4	0.8160 (3)	−0.0204 (2)	1.0670 (6)	0.0723 (11)	
H4	0.8010	−0.0602	1.1479	0.087*	
C5	0.8807 (3)	0.0460 (2)	1.1294 (4)	0.0660 (10)	
H5	0.9114	0.0505	1.2506	0.079*	
C6	0.8996 (3)	0.10584 (17)	1.0104 (4)	0.0516 (8)	
H6	0.9425	0.1515	1.0508	0.062*	
C7	0.5726 (3)	0.34525 (15)	0.7167 (4)	0.0500 (8)	
H7A	0.6031	0.3497	0.8423	0.075*	
H7B	0.4948	0.3718	0.6935	0.075*	
H7C	0.6298	0.3694	0.6462	0.075*	
C8	0.5571 (2)	0.25952 (15)	0.6673 (3)	0.0361 (7)	
C9	0.4326 (2)	0.22753 (15)	0.6112 (3)	0.0370 (7)	
C10	0.3985 (3)	0.14763 (16)	0.6207 (4)	0.0427 (7)	
C11	0.2074 (3)	0.17989 (19)	0.4865 (4)	0.0581 (9)	
H11	0.1286	0.1650	0.4380	0.070*	
C12	0.2400 (3)	0.2581 (2)	0.4845 (4)	0.0588 (9)	
H12	0.1817	0.2953	0.4381	0.071*	
C13	0.4793 (3)	0.08310 (16)	0.7054 (4)	0.0641 (10)	
H13A	0.4296	0.0395	0.7368	0.096*	
H13B	0.5272	0.1031	0.8121	0.096*	
H13C	0.5329	0.0651	0.6220	0.096*	

### Atomic displacement parameters (Å^2^)

**Table:**

	*U*^11^	*U*^22^	*U*^33^	*U*^12^	*U*^13^	*U*^23^
N1	0.0289 (14)	0.0326 (12)	0.0566 (16)	−0.0015 (10)	−0.0026 (11)	−0.0017 (11)
N2	0.0279 (14)	0.0445 (13)	0.0413 (15)	−0.0002 (11)	−0.0037 (11)	−0.0015 (11)
N3	0.0346 (16)	0.0528 (15)	0.0610 (18)	0.0029 (12)	−0.0043 (13)	0.0027 (13)
N4	0.0402 (17)	0.0594 (16)	0.0645 (18)	−0.0082 (13)	0.0006 (13)	−0.0014 (14)
O1	0.0291 (12)	0.0601 (13)	0.0667 (14)	−0.0069 (9)	−0.0033 (10)	0.0065 (11)
O2	0.0515 (13)	0.0615 (13)	0.0383 (12)	0.0083 (10)	0.0008 (9)	−0.0039 (10)
S1	0.0308 (4)	0.0479 (4)	0.0439 (5)	0.0027 (3)	0.0003 (3)	0.0018 (4)
C1	0.0321 (17)	0.0405 (16)	0.0429 (19)	0.0085 (12)	0.0042 (13)	−0.0010 (14)
C2	0.044 (2)	0.0517 (19)	0.063 (2)	0.0042 (15)	−0.0036 (16)	0.0007 (18)
C3	0.065 (2)	0.049 (2)	0.096 (3)	−0.0003 (16)	0.007 (2)	0.013 (2)
C4	0.080 (3)	0.057 (2)	0.086 (3)	0.019 (2)	0.037 (2)	0.025 (2)
C5	0.085 (3)	0.069 (2)	0.047 (2)	0.034 (2)	0.0166 (19)	0.004 (2)
C6	0.061 (2)	0.0464 (18)	0.048 (2)	0.0134 (15)	0.0079 (16)	−0.0043 (16)
C7	0.0441 (19)	0.0471 (18)	0.056 (2)	0.0026 (13)	−0.0027 (15)	−0.0038 (15)
C8	0.0340 (17)	0.0413 (16)	0.0323 (17)	0.0031 (13)	0.0014 (13)	0.0011 (13)
C9	0.0316 (17)	0.0447 (17)	0.0342 (17)	0.0030 (13)	0.0019 (13)	−0.0008 (14)
C10	0.0374 (18)	0.0484 (17)	0.0422 (18)	−0.0012 (14)	0.0045 (14)	−0.0009 (15)
C11	0.0324 (19)	0.073 (2)	0.066 (2)	−0.0047 (17)	−0.0047 (16)	−0.0056 (19)
C12	0.032 (2)	0.069 (2)	0.071 (2)	0.0059 (16)	−0.0084 (16)	0.0003 (18)
C13	0.050 (2)	0.0490 (19)	0.090 (3)	−0.0034 (15)	−0.0032 (18)	0.0134 (19)

### Geometric parameters (Å, °)

**Table:**

N1—N2	1.387 (3)	C4—H4	0.9300
N1—S1	1.628 (2)	C5—C6	1.376 (4)
N1—H1	0.8600	C5—H5	0.9300
N2—C8	1.277 (3)	C6—H6	0.9300
N3—C12	1.316 (3)	C7—C8	1.491 (3)
N3—C9	1.337 (3)	C7—H7A	0.9600
N4—C11	1.323 (3)	C7—H7B	0.9600
N4—C10	1.334 (3)	C7—H7C	0.9600
O1—S1	1.4163 (18)	C8—C9	1.480 (3)
O2—S1	1.4220 (19)	C9—C10	1.397 (3)
S1—C1	1.740 (3)	C10—C13	1.490 (3)
C1—C6	1.370 (4)	C11—C12	1.361 (4)
C1—C2	1.377 (3)	C11—H11	0.9300
C2—C3	1.378 (4)	C12—H12	0.9300
C2—H2	0.9300	C13—H13A	0.9600
C3—C4	1.359 (5)	C13—H13B	0.9600
C3—H3A	0.9300	C13—H13C	0.9600
C4—C5	1.372 (4)		
			
N2—N1—S1	114.60 (16)	C5—C6—H6	120.2
N2—N1—H1	122.7	C8—C7—H7A	109.5
S1—N1—H1	122.7	C8—C7—H7B	109.5
C8—N2—N1	117.3 (2)	H7A—C7—H7B	109.5
C12—N3—C9	117.2 (2)	C8—C7—H7C	109.5
C11—N4—C10	117.8 (3)	H7A—C7—H7C	109.5
O1—S1—O2	120.49 (13)	H7B—C7—H7C	109.5
O1—S1—N1	103.92 (12)	N2—C8—C9	116.0 (2)
O2—S1—N1	106.70 (11)	N2—C8—C7	124.3 (2)
O1—S1—C1	109.44 (12)	C9—C8—C7	119.5 (2)
O2—S1—C1	107.89 (12)	N3—C9—C10	120.9 (2)
N1—S1—C1	107.71 (12)	N3—C9—C8	113.8 (2)
C6—C1—C2	121.1 (3)	C10—C9—C8	125.3 (2)
C6—C1—S1	119.1 (2)	N4—C10—C9	120.2 (2)
C2—C1—S1	119.7 (2)	N4—C10—C13	115.0 (2)
C1—C2—C3	118.9 (3)	C9—C10—C13	124.8 (2)
C1—C2—H2	120.6	N4—C11—C12	121.5 (3)
C3—C2—H2	120.6	N4—C11—H11	119.2
C4—C3—C2	119.8 (3)	C12—C11—H11	119.2
C4—C3—H3A	120.1	N3—C12—C11	122.3 (3)
C2—C3—H3A	120.1	N3—C12—H12	118.9
C3—C4—C5	121.4 (3)	C11—C12—H12	118.9
C3—C4—H4	119.3	C10—C13—H13A	109.5
C5—C4—H4	119.3	C10—C13—H13B	109.5
C4—C5—C6	119.2 (3)	H13A—C13—H13B	109.5
C4—C5—H5	120.4	C10—C13—H13C	109.5
C6—C5—H5	120.4	H13A—C13—H13C	109.5
C1—C6—C5	119.5 (3)	H13B—C13—H13C	109.5
C1—C6—H6	120.2		
			
S1—N1—N2—C8	−178.3 (2)	N1—N2—C8—C9	177.5 (2)
N2—N1—S1—O1	−177.34 (18)	N1—N2—C8—C7	1.7 (4)
N2—N1—S1—O2	54.3 (2)	C12—N3—C9—C10	−2.9 (4)
N2—N1—S1—C1	−61.3 (2)	C12—N3—C9—C8	176.5 (3)
O1—S1—C1—C6	30.8 (3)	N2—C8—C9—N3	−152.2 (3)
O2—S1—C1—C6	163.6 (2)	C7—C8—C9—N3	23.8 (4)
N1—S1—C1—C6	−81.6 (2)	N2—C8—C9—C10	27.2 (4)
O1—S1—C1—C2	−151.7 (2)	C7—C8—C9—C10	−156.8 (3)
O2—S1—C1—C2	−18.9 (3)	C11—N4—C10—C9	−1.1 (4)
N1—S1—C1—C2	96.0 (2)	C11—N4—C10—C13	177.0 (3)
C6—C1—C2—C3	0.9 (4)	N3—C9—C10—N4	3.5 (4)
S1—C1—C2—C3	−176.5 (2)	C8—C9—C10—N4	−175.9 (3)
C1—C2—C3—C4	0.3 (5)	N3—C9—C10—C13	−174.4 (3)
C2—C3—C4—C5	−1.8 (5)	C8—C9—C10—C13	6.3 (5)
C3—C4—C5—C6	2.0 (5)	C10—N4—C11—C12	−1.6 (5)
C2—C1—C6—C5	−0.7 (4)	C9—N3—C12—C11	0.2 (5)
S1—C1—C6—C5	176.8 (2)	N4—C11—C12—N3	2.1 (5)
C4—C5—C6—C1	−0.7 (4)		

### Hydrogen-bond geometry (Å, °)

**Table:**

*D*—H···*A*	*D*—H	H···*A*	*D*···*A*	*D*—H···*A*
N1—H1···O2^i^	0.86	2.34	3.027 (3)	137

Symmetry codes: (i) *x*, −*y*+1/2, *z*+1/2.
